# Significance of Lipid-Derived Reactive Aldehyde-Specific Immune Complexes in Systemic Lupus Erythematosus

**DOI:** 10.1371/journal.pone.0164739

**Published:** 2016-10-17

**Authors:** Gangduo Wang, Silvia S. Pierangeli, Rohan Willis, Emilio B. Gonzalez, Michelle Petri, M. Firoze Khan

**Affiliations:** 1 Department of Pathology, University of Texas Medical Branch, Galveston, TX, United States of America; 2 Department of Internal Medicine, Division of Rheumatology, University of Texas Medical Branch, Galveston, TX, United States of America; 3 Division of Rheumatology, Department of Medicine, Johns Hopkins University School of Medicine, Baltimore, MD, United States of America; Instituto Nacional de Ciencias Medicas y Nutricion Salvador Zubiran, MEXICO

## Abstract

Even though systemic lupus erythematosus (SLE) is associated with high morbidity and mortality rates among young and middle-aged women, the molecular mechanisms of disease pathogenesis are not fully understood. Previous studies from our laboratory suggested an association between oxidative stress and SLE disease activity (SLEDAI). To further assess the role of reactive oxygen species (ROS) in SLE, we examined the contribution of lipid-derived reactive aldehydes (LDRAs)-specific immune complexes in SLE. Sera from 60 SLE patients with varying SLEDAI and 32 age- and gender- matched healthy controls were analyzed for oxidative stress and related markers. Patients were divided into two groups based on their SLEDAI scores (<6 and ≥ 6). Both SLEDAI groups showed higher serum 4-hydroxynonenal (HNE)-/malondialdehyde (MDA)-protein adducts and their specific immune complexes (HNE-/MDA-specific ICs) together with IL-17 than the controls, but the levels were significantly greater in the high SLEDAI (≥ 6) group. Moreover, the serum levels of anti-oxidant enzymes Cu/Zn superoxide dismutase (SOD) and catalase (CAT) were significantly reduced in both patient groups compared to controls. Remarkably, for the first time, our data show that increased HNE-/MDA-specific ICs are positively associated with SLEDAI and elevated circulating immune complexes (CICs), suggesting a possible causal relationship among oxidative stress, LDRA-specific ICs and the development of SLE. Our findings, apart from providing firm support to an association between oxidative stress and SLE, also suggest that these oxidative stress markers, especially the HNE-/MDA-specific ICs, may be useful in evaluating the prognosis of SLE as well as in elucidating the mechanisms of disease pathogenesis.

## Introduction

Autoimmune diseases (ADs) include approximately 80 different disorders affecting ~ 5% of the population [1, 2]. Systemic lupus erythematosus (SLE) is a chronic AD, characterized by the production of autoantibodies and formation of immune complexes, which trigger inflammation and lead to tissue destruction, multi-system organ dysfunction and premature mortality [3–5]. Even though multiple factors, including genetic, hormonal and environmental triggers, are thought to contribute to the development of a systemic immune response to self, the molecular mechanisms and pathogenesis underlying this autoimmune response are not completely understood.

There has been growing evidence to suggest that oxidative stress has a role in the pathogenesis of ADs including SLE [6–9]. Indeed, excessive oxidative stress and decreased antioxidant levels are reported in various ADs, particularly SLE [6, 7, 9, 10], and the enhanced oxidative stress has been linked to disease induction, progression and severity of SLE [6, 11–13]. Moreover, antioxidants such as N-acetylcysteine (NAC) can improve autoimmune response and reduce SLE disease activity by decreasing oxidative stress, further supporting the significance of oxidative stress in disease pathogenesis [9, 10, 14, 15].

It has been widely accepted that excessive reactive oxygen species (ROS) production has the potential to damage macromolecules, including lipids, proteins and DNA [11, 12, 16–19]. The primary targets of ROS are double bonds in polyunsaturated fatty acids in the cell membrane, resulting in increased lipid peroxidation and eventually leading to the formation of reactive aldehydes. These lipid-derived reactive aldehydes (LDRAs) such as 4-hydroxynonenal (4-HNE) and malondialdehyde (MDA) are highly electrophilic and can bind covalently to proteins resulting in their structural modifications and affecting biological functions [16, 18–23]. In recent years, several lines of evidence have shown elevated formation of HNE- and MDA-modified proteins in SLE, and also demonstrated that those modified proteins are immunogenic and associated with SLE disease activity [6, 7, 16, 18, 20, 23]. Even though LDRAs have been implicated in SLE, the potential of LDRAs in eliciting an autoimmune response and contribution to disease pathogenesis and progression remains to be fully elucidated.

Formation of circulating immune complexes (CICs) is not only one of the major characteristics of SLE, but they also play a crucial role in the pathogenesis of SLE via affecting immune response and inducing cells to release tissue damaging substances [3–5]. Therefore, to further evaluate the significance of oxidative stress, especially LDRAs, in SLE progression and pathogenesis, we examined the levels of HNE-/MDA-protein adducts, superoxide dismutase (SOD) and catalase (CAT), CICs and IL-17 in the sera of SLE patients. More importantly, for the first time, we also present data on serum HNE-/MDA-specific immune complexes (ICs) and their relationship with SLE disease activity index (SLEDAI) or serum CICs in human samples. Our findings, apart from providing a firm support to a strong connection between LDRA-specific ICs and SLE, also suggest a causative role of LDRAs in SLE.

## Materials and Methods

### Patients and healthy donors

A total of 60 SLE patients (53 females and 7 males), as defined by the Systemic Lupus International Collaborating Clinics (SLICC) SLE Classification Criteria [24], and the age range 22–73 years (45.8 ± 11.9) were recruited in this study. The Safety of Estrogens in Lupus Erythematosus National Assessment (SELENA) version of the SLE Disease Activity Index (SLEDAI) was determined using the SLE Disease Activity Measure [25], and the SLEDAI scores among SLE patients ranged from 0–30 (7.5 ± 6.9). These SLE patients were divided into two groups based on the SLEDAI: low SLEDAI group comprised 28 SLE patients (25 females and 3 males) with SLEDAI < 6, age range 22–62 years (47.5 ± 10.9), and high SLEDAI group comprised 32 SLE patients (28 females and 4 males) with SLEDAI ≥ 6 and, age range 23–73 years (44.5 ± 12.7). The control group comprised 32 healthy subjects (29 females and 3 males) with age range 27–67 years (41.3 ± 11.3). Mean ages of the two SLE subgroups and the normal control group were not statistically different. The racial/ethnic and gender composition of the SLE groups were comparable to the control group. The study was approved by University of Texas Medical Branch Institutional Review Board (IRB, No. 07–262). After obtaining informed consent from patients and controls, blood samples were collected in sterilized vacuum tubes and the harvested serum from individual subjects was stored in small aliquots at -80°C until analysis.

### Quantification of serum HNE-/MDA-protein adducts

Serum HNE-/MDA-protein adducts in the SLE patients and controls were quantitated using competitive ELISAs as described in our previous studies [15, 26, 27]. Briefly, to perform the competitive ELISA assay, the standards and human samples were incubated with rabbit anti-HNE (1:3000 diluted) or anti-MDA (1:2000 diluted) sera (Alpha Diagnostics, San Antonio, TX) overnight at 4°C. HNE-/MDA-ovalbumin adducts or ovalbumin (0.5 μg/well) coated plates were blocked with a blocking buffer [tris-buffered saline (TBS) containing 1% BSA, Sigma, St. Louis, MO], then 50μl/well of each of the above mentioned mixtures were added and incubated for 2h at RT. After washing, 50 μl/well of goat anti-rabbit IgG-HRP (1:2000 diluted, Millpore, Billerica, MA) was added and incubated for 1h at RT. Following washing, 100 μl/well of TMB peroxidase substrate (KPL, Gaithersburg, MD) was added. After the reaction was stopped by 100 μl/well of 2 M H_2_SO_4_, the absorbance was determined at 450 nm by using a Bio-Rad Benchmark plus microplate spectrophotometer (Bio-Rad Laboratories, Hercules, CA).

### Determination of HNE-/MDA-specific ICs in the sera

The immune complexes of HNE-/MDA-protein adducts with their respective specific antibodies in the sera of SLE patients and controls were analyzed using specific ELISAs established in our laboratory [28]. Briefly, 96-well plates were coated with 100 μl/well of rabbit anti-HNE- or anti-MDA-protein adduct antibodies (1:1000 diluted; Alpha Diagnostics Int’l) overnight at 4°C. After blocking with 200 μl/well blocking buffer (Sigma), the coated plates were added and incubated with 100 μl/well of diluted serum samples at RT for 2 h. The plates were extensively washed with TBST and incubated with 100 μl/well of rabbit anti-human IgG-HRP at RT for 1 h. After extensive washing, the plates were added with 100 μl/well of TMB substrate, followed by addition of 100 μl/well of 2M H_2_SO_4_ 10 min later to stop the reaction. Finally, the OD at 450 nm was determined using a Bio-Rad Benchmark plus microplate spectrophotometer (Bio-Rad Laboratories).

### Quantitation of serum superoxide dismutase and catalase

The contents of Cu/Zn SOD and CAT in the sera were quantitated using the Cu/Zn superoxide dismutase ELISA kit (Bender MedSystems, Burlingame, CA) and a catalase Human ELISA kit (Abcam, Cambridge, MA), respectively, following manufacturer’s manual.

### Quantification of CICs in the sera

Serum levels of CICs were quantitated by using CIC-C3d human ELISA kits (Abcam) following manufacturer’s manual. Briefly, all reagents including standards, controls and samples were prepared following manufacturer’s protocol. One hundred μl of standards, controls and samples (all duplicates) were added into their respective wells (two wells left for substrate blank) and incubated at RT for 2h. The wells were extensively washed with washing solution and 100 μl/well of diluted CIC-C3d anti-IgG HRP were added. After incubation and extensive washing, 100 μl TMB substrate solution was added into all wells and incubated for 15 min at RT in the dark. This was followed by addition of 100 μl stop solution into each well to stop the reaction. The absorbance was measured at 450 nm using a Bio-Rad Benchmark plus microplate spectrophotometer (Bio-Rad Laboratories).

### Measurement of serum IL-17A

The levels of IL-17 in the sera were assessed by using an ELISA kit for IL-17A (Thermo Fisher Scientific, Waltham, MA). Assays were carried out according to the manufacturer’s instructions.

### Statistical analysis

All data were analyzed using GraphPad Instat 3 software (La Jolla, CA). One-way ANOVA and Tukey-Kramer multiple comparisons test was used to compare data among the groups made by *p* value determination. Spearman’s rank correlation was used to calculate correlation coefficients between serum HNE-MDA-specific ICs and SLEDAI scores or CICs. The *p* values < 0.05 were considered to be statistically significant. The values are presented as means ± SD.

## Results

### The formation of LDRA-protein adducts in the sera of SLE patients

**Fig 1:**
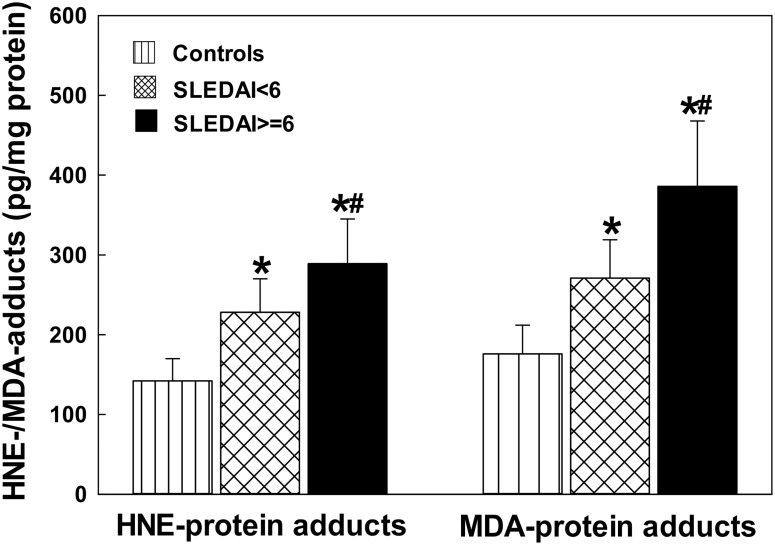
HNE-and MDA-protein adducts in the sera of SLE patients with SLEDAI < 6 (n = 28) and SLEDAI ≥ 6 (SLEDAI> = 6 in the figure; n = 32) vs. controls (n = 32). The results are means ± SD. * p < 0.05 vs. controls; # p < 0.05 vs. SLEDAI < 6 patients.

LDRAs such as HNE and MDA have been implicated in the pathogenesis of SLE [6, 7, 16, 18, 20, 23]. However, the role of LDRAs in the initiation and development of SLE is largely unclear. To evaluate the involvement of LDRAs in the pathogenesis of SLE, we first determined the formation of HNE-/MDA-protein adducts in the sera of SLE patients vs. age- and gender-matched controls, because HNE and MDA are two major LDRAs and generally accepted biomarkers of oxidative stress/lipid peroxidation [6, 8, 15, 16, 18, 20, 27]. As evident from Fig 1, there was significantly increased formation of HNE-/MDA-protein adducts in SLE patients, both SLEDAI < 6 and SLEDAI ≥ 6 groups, as compared to the controls (p < 0.05), suggesting increased lipid peroxidation/oxidative stress in SLE patients. Remarkably, increases in the HNE- and MDA-protein adducts in the patients with SLEDAI ≥ 6 were also greater compared with the patients with SLEDAI < 6 (p < 0.05), suggesting a positive relation between the elevated LDRAs and SLEDAI.

### The levels of LDRA-specific ICs in the sera of SLE patients

**Fig 2:**
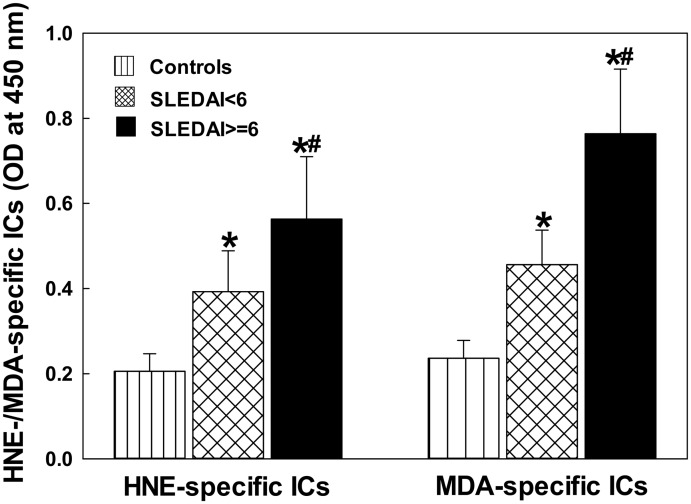
Serum HNE- and MDA-specific ICs in SLE patients with SLEDAI < 6 (n = 28) and SLEDAI ≥ 6 (SLEDAI> = 6 in the figure; n = 32) vs. controls (n = 32). The results are means ± SD. * p < 0.05 vs. controls; # p < 0.05 vs. SLEDAI < 6 patients.

To validate the importance of LDRAs in the disease pathogenesis of SLE, we next determined the formation of serum HNE-/MDA-specific ICs as a function of SLEDAI (Fig 2). The SLE patients, both SLEDAI < 6 and SLEDAI ≥ 6, were found to have significantly higher levels of HNE-specific ICs in comparison to the controls (p < 0.05). Interestingly, the increases in the formation of HNE-specific ICs were significantly greater in patients with SLEDAI ≥ 6 compared to the patients with SLEDAI < 6 (p < 0.05). Similarly, the formation of serum MDA-specific ICs was also elevated in SLE patients in both SLEDAI < 6 and SLEDAI ≥ 6 groups, and even much greater in patients with SLEDAI ≥ 6 vs. SLEDAI < 6 (p < 0.05). Remarkable increases in the formation of serum LDRA-specific ICs and even greater LDRA-specific ICs in SLE patients with higher SLEDAI (≥ 6), suggest that increased LDRA-modified proteins are immunogenic and strongly associated with the progression of the disease activity.

### Correlation of serum LDRA-specific ICs with SLEDAI

**Fig 3:**
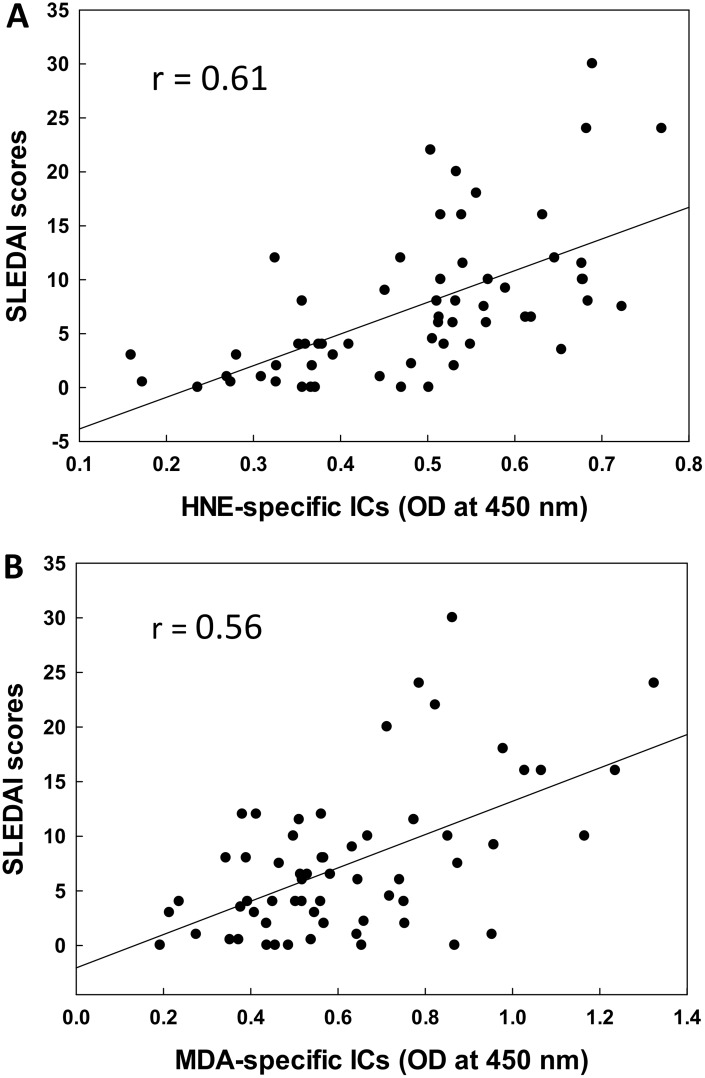
Correlation of serum HNE-specific ICs (A) or MDA-specific ICs (B) with SLEDAI scores. The correlation was established by calculating correlation coefficients between HNE- or MDA-specific ICs and SLEDAI scores.

To further determine the potential contribution of LDRAs in SLE, we analyzed the relationship of the increases in serum HNE-/MDA-specific ICs with SLEDAI (Fig 3). Our data clearly show a significant correlation between HNE-specific ICs and SLEDAI (r = 0.61, p < 0.01; Fig 3A). Similarly, a remarkable correlation between MDA-specific ICs and SLEDAI (r = 0.55, p < 0.01; Fig 3B) was also observed. These results further support the potential role of LDRAs in SLE, and also suggest that the HNE-/MDA-specific ICs may be useful in predicting the progression of SLE.

### Serum levels of Cu/Zn SOD and CAT activity in the SLE patients

The markers of increased oxidative stress and impaired antioxidant capacity have been shown to be significantly correlated with disease activity of SLE [5, 6, 11, 18]. To assess the redox status in SLE patients, we measured the serum activity of Cu/Zn SOD and CAT, two major antioxidant enzymes. There were significant decreases in Cu/Zn SOD and CAT activities in the SLE patients, both in SLEDAI < 6 and SLEDAI ≥ 6 groups, compared to the controls (p < 0.05). Furthermore, much lower SOD activity was observed in the SLE patients with SLEDAI ≥ 6 in comparison to SLE patients with SLEDAI < 6 (p < 0.05, Table 1). The results of remarkable reduction in serum Cu/Zn SOD and CAT activities provide further proof of a compromised antioxidant balance as also reported earlier [5, 11].

**Table 1:** Serum levels of SOD, CAT and CIC4 in controls and SLE patients.

	Controls	SLEDAI < 6	SLEDAI ≥ 6
SOD (ng/ml)	62.5 ±12.5	43.6±10.6*	31.2 ± 8.5*^#^
CAT (ng/ml)	35.7 ±6.8	23.8±6.1*	20.2±5.7*
CICs (μg/ml)	212.4±58.5	398.2±157.4*	621.9±215.1*^#^

Note: The results are means ± SD.

* p<0.05 vs. controls;

^#^ p<0.05 vs. SLEDAI<6 group.

### CICs concentration in the sera of SLE patients

SLE is considered as a prototype immune complex disease with immune complex formation and subsequent complement activation playing a vital role in the pathogenesis of SLE [3–5]. Therefore, we determined the CICs concentration in the serum of SLE patients. As shown in Table 1, we observed a significant increase in CICs levels in the SLE patients, both with SLEDAI < 6 and SLEDAI ≥ 6, compared to the controls (p < 0.05). However, remarkably higher CICs concentration was found in the SLE patients with SLEDAI ≥ 6 compared to the SLE patients with SLEDAI < 6 (p < 0.05, Table 1).

### Correlation of serum LDRA-specific ICs with CICs

**Fig 4:**
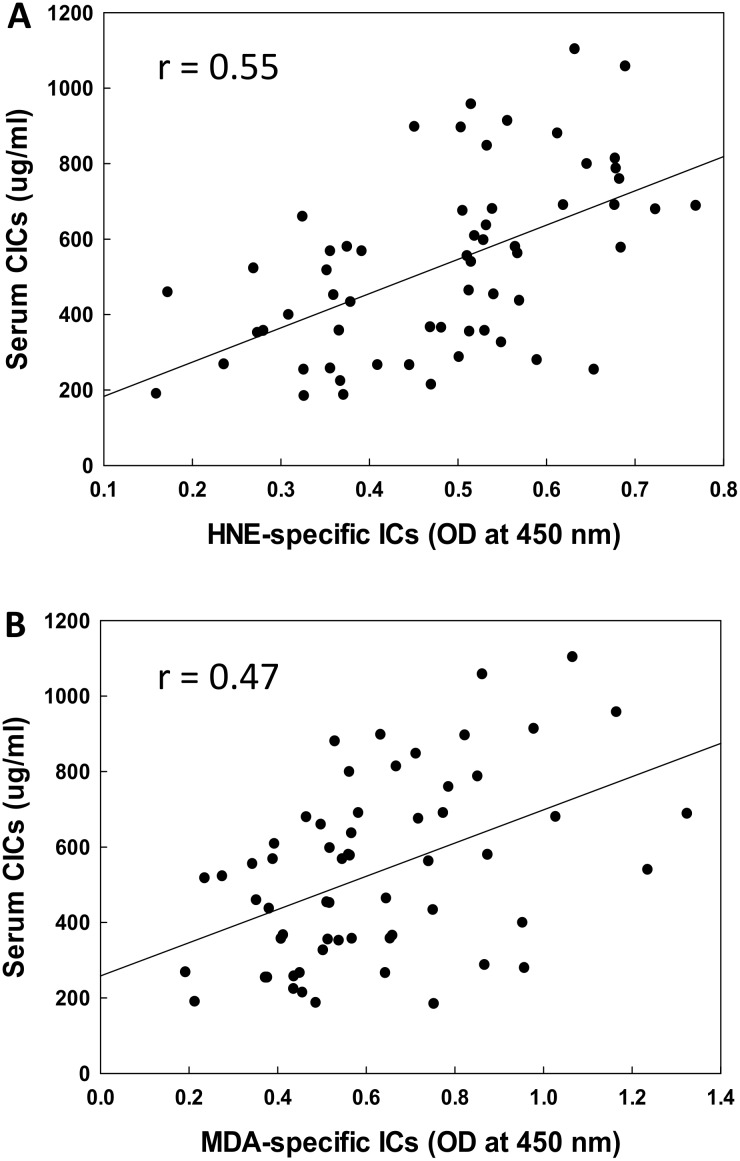
Correlation of serum HNE-specific ICs (A) or MDA-specific ICs (B) with serum CICs. The correlation was established by calculating correlation coefficients between HNE- or MDA-specific ICs and serum CICs.

To further evaluate the potential role of LDRAs in SLE, we also explored the possible relationship of the increases in serum HNE-/MDA-specific ICs with CICs (Fig 4). We observed a significantly positive correlation between HNE-specific ICs and CICs (r = 0.52, p < 0.01; Fig 4A). Furthermore, a strong correlation between MDA-specific ICs and CICs (r = 0.48, p < 0.01; Fig 4B) was also observed. These results suggest that increases in HNE-/MDA-specific ICs could be contributing to increased total CICs, and LDRAs may play a possible causal role in SLE via triggering the autoimmune response.

### Serum IL-17 levels in the SLE patients

**Fig 5:**
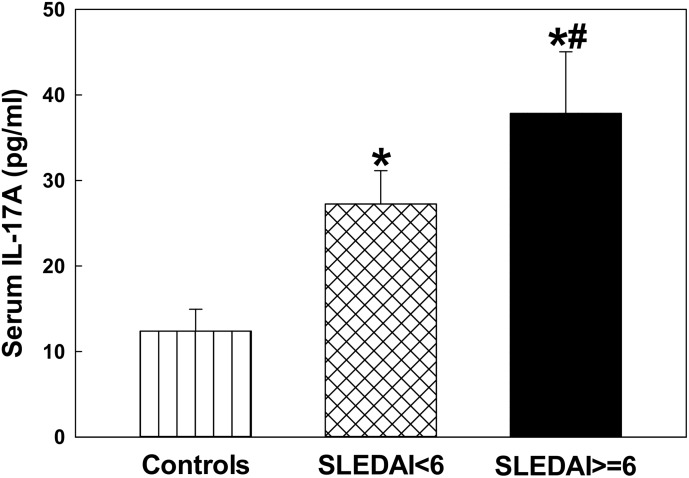
IL-17A levels in the sera of SLE patients with SLEDAI < 6 (n = 28) and SLEDAI ≥ 6 (SLEDAI> = 6 in the figure; n = 32) vs. controls (n = 32). Values are means ± SD. * p < 0.05 vs. controls; # p < 0.05 vs. SLEDAI < 6 patients.

**Fig 6:**
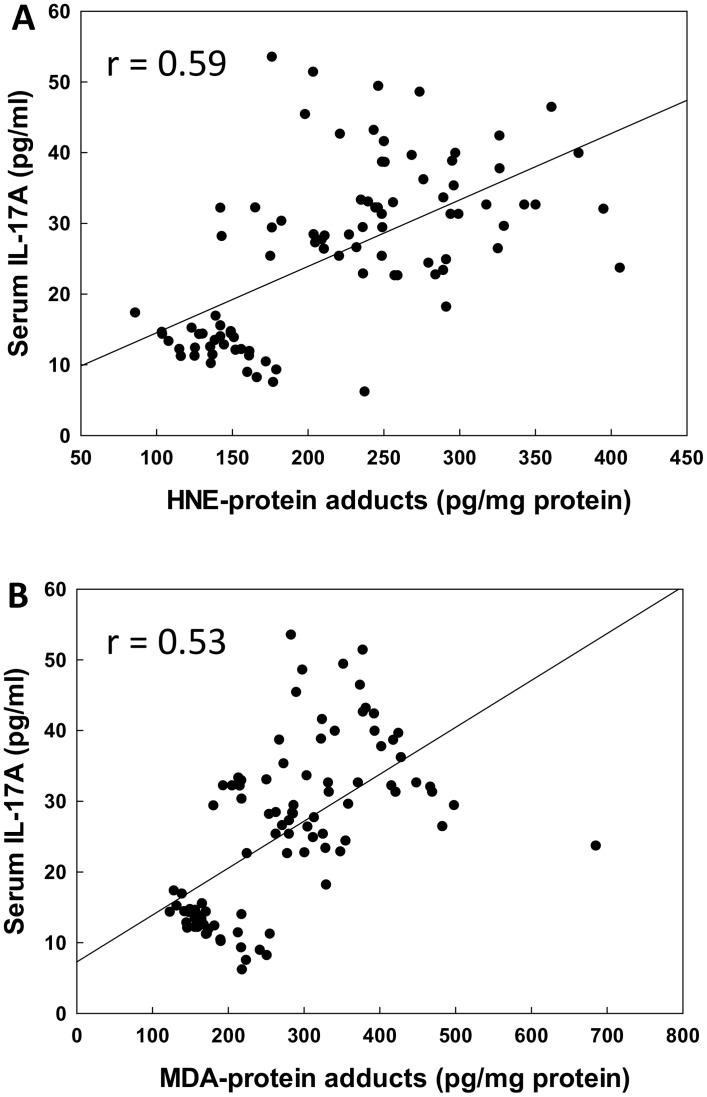
Correlation of serum HNE-protein adducts (A) or MDA-protein adducts (B) with IL-17A. The correlation was established by calculating correlation coefficients between serum HNE- or MDA-protein adducts and IL-17A.

Increasing evidence suggests that IL-17 and Th17 cells may be involved in the pathogenesis of SLE [29–32]. However, the functional role of IL-17 in the immunopathogenesis of SLE remains largely unknown. To provide additional evidence supporting our hypothesis that LDRAs mediate an autoimmune response via activation of T lymphocytes, particularly Th17 cells, the serum levels of IL-17 in the SLE patients was determined. As evident from Fig 5, the levels of IL-17 were statistically higher in the SLE patients, both in SLEDAI < 6 and SLEDAI ≥ 6 groups (p < 0.05), in comparison to the controls. However, the increases in IL-17 were remarkably greater in the SLEDAI ≥ 6 group and also significantly higher than the SLEDAI <6 group (p < 0.05), suggesting a positive correlation with SLEDAI. Moreover, the increased serum levels of IL-17 also had a strongly positive correlation with increased levels of HNE-/MDA-protein adducts (r = 0.59 and 0.53 for HNE-protein adducts and MDA-protein adducts, respectively, p < 0.05, Fig 6).

## Discussion

SLE is one of most extensively studied ADs characterized by increased generation of autoantibodies and formation of immune complexes [3–6, 9, 12]. However, the etiology of the disease, especially initial immunizing self-antigens that drive the development of SLE, is largely unclear. In an attempt to provide novel evidence and validate our central hypothesis that initiation of autoimmunity may be mediated by increased formation of HNE-/MDA-modified proteins following excessive ROS generation and oxidative stress, oxidative stress markers and related indexes were quantitated in the sera of SLE patients vs. age- and gender-matched control subjects. Our results show increased prevalence of both HNE-/MDA-protein adducts and their specific ICs together with decreases in SOD and CAT in SLE patients. More interestingly, for the first time, our study shows that increased HNE-/MDA-specific ICs are very positively associated with SLEDAI and enhanced CICs in SLE patients, suggesting a possible causal role of LDRAs in the pathogenesis and/or progression of SLE.

Although it is believed that oxidative stress has been involved in the pathogenesis of SLE [6, 8, 21, 22, 33, 34], the mechanisms of its involvement in the initiation and progression of SLE remains largely unknown. Earlier studies showed that LDRA-modified proteins induced strong T-cell dependent antibody response and triggered ADs, suggesting the breaking of immunological tolerance by LDRA-protein adducts [8, 33, 34]. Previously, we reported a good correlation between serum anti-MDA-/HNE-protein adduct antibodies and SLEDAI scores, supporting the potential role of LDRAs in the SLE progression [6].

The Uchida research group proposed the idea of molecular mimicry between LDRA-protein adducts and modified- or native-DNA following sequence and cross-reaction analysis between anti-LDRA-modified protein and anti-DNA antibodies [21, 22]. In this study, when the SLE patients were divided into two groups based on their SLEDAI (<6 and ≥ 6), both groups showed higher serum HNE-/MDA-protein adducts and HNE-/MDA-specific ICs than the controls, but the levels were significantly greater in the high SLEDAI (≥ 6) group, suggesting an ongoing involvement of LDRAs in SLE activity. In addition, a highly positive correlation between serum HNE-/MDA-specific ICs and SLEDAI observed in the current study not only confirms a strong association among generation of HNE/MDA, formation of HNE/MDA-protein adducts and SLE disease activity, but also suggests that the HNE/MDA-protein adducts are immunogenic and actively form LDRA-specific ICs contributing to the progression of SLE. More importantly, the strongly positive correlation between serum HNE-/MDA-specific ICs and serum CICs shows a potential molecular mimicry between the HNE-/MDA-protein adducts and DNA (modified or native form).

The remarkable relation also suggests that HNE-/MDA-specific ICs could be a source of increased total CICs via a possible production of anti-DNA autoantibodies in response to LDRA-modified self-proteins (self-antigens) [18, 21, 22]. Immune complex formation and subsequent complement activation are characteristics, key mediators and disease predictors of SLE, and also play a crucial role in the pathogenesis of SLE [3, 28, 35]. Excessive amounts of CICs can form tissue/organ IC deposits, especially in the kidney, and then cause severe end-tissue/organ damage via triggering release of inflammatory mediators including complement and cytokines, activating complement and inducing immune dysfunction. Thus immune complexes could play a central pathogenic role in causing tissue damage in the disease [3, 35, 36]. Therefore, the findings in this study apart from supporting that these LDRA-ICs could be valuable in evaluating the progression of the disease, also suggest a possible causal relationship among oxidative stress, HNE-/MDA-specific ICs and development of SLE. Further characterization of the biological consequence of the production of these LDRA-specific ICs would be an important forward step.

Under physiological conditions, the adverse effects of ROS are normally controlled by the antioxidant defense mechanisms including enzymatic and non-enzymatic antioxidant defense systems [5, 6, 11, 17]. SOD and CAT are two major antioxidant defense enzymes and the first line of defense against ROS. They scavenge the ROS production via catalyzing the dismutation of the superoxide radical (O_2_^**.-**^) by SOD into hydrogen peroxide (H_2_O_2_) which is further converted into water and molecular oxygen by CAT, and thereby maintaining an appropriate cellular redox balance [5, 17, 19]. The findings of increased LDRAs in SLE patients observed in this study led us to evaluate the state of antioxidant defense enzymes SOD and CAT in these subjects. The activity of Cu/Zn SOD and CAT were remarkably lower in the SLE patients, particularly in the higher SLEDAI group. The reduced activity of Cu/Zn SOD and CAT along with elevated LDRAs confirm an alteration in redox balance leading to enhanced oxidative stress in these SLE patients [5, 17].

It is generally accepted that T cells contribute to the initiation and the development of ADs including SLE [29–32, 37]. Activated CD4+ T cells differentiate into at least three subgroups, Th1, Th2 and Th17, according to their distinct cytokine secretions and functions. Th17 cells, which produce IL-17, IL-21and IL-22, appear to be key effector T cells in a variety of human ADs, including SLE [29, 31, 32, 38]. IL-17 is a proinflammatory cytokine produced by activated T cells, particularly Th17 cells, and plays an important role in disease progression and pathogenesis of SLE [29, 31, 32, 37, 38]. Previous studies in our laboratory demonstrated that LDRA-modified proteins contribute to autoimmunity via activating TH17 cells in an animal model [26]. In the current study, we indeed observed a SLEDAI-related increases in IL-17 levels, and also observed a strongly positive relationship between increased LDRA-modified proteins and elevated serum IL-17. Therefore, our findings strongly support the potential role of IL-17 in the disease pathogenesis of SLE and further suggest that the HNE-/MDA-protein adducts may contribute to initiation and/or development of SLE via activating TH17 cells.

In conclusion, our results clearly show an imbalance between oxidative stress and antioxidant defense enzymes in SLE patients. The increased formation of HNE-/MDA-protein adducts and LDRA-specific ICs observed in this study provide evidence that LDRA-modified endogenous proteins are immunogenic and could break self-tolerance and/or elicit an autoimmune response by stimulating T cells (especially Th17 cells). Importantly, for the first time, we provide an evidence for a strong association between HNE-/MDA-specific ICs and SLEDAI, and between HNE-/MDA-specific ICs and elevated CICs in the human samples. These findings support that oxidative stress markers, especially LDRA-specific-ICs, could be useful in evaluating SLE disease activity/progression of the disease, and also suggest that LDRA-modified proteins may play a causative role in SLE pathogenesis. It would be important, however, to include greater sample size for both genders in future studies. Furthermore, longitudinal studies and characterization of autoantigens/autoantibodies comprising the CICs are necessary to further establish the role of LDRAs as a contributing pathogenic mechanism in SLE, and to assess the usefulness of LDRAs in evaluating disease progression, as well as in developing therapeutic strategies for SLE.
